# Role of Removed Lymph Nodes on the Prognosis of M0 Small-Bowel Neuroendocrine Tumors: a Propensity Score Matching Analysis from SEER Database

**DOI:** 10.1007/s11605-021-04994-3

**Published:** 2021-06-09

**Authors:** Jie-bin Xie, Yue-shan Pang, Xun Li, Xiao-ting Wu

**Affiliations:** 1 Department of Gastrointestinal Surgery, West China Hospital, Sichuan University, Chengdu, 610041 Sichuan China; 2 Department of Gastrointestinal Surgery, Affiliated Hospital of North Sichuan Medical College, Nanchong, Sichuan, China; 3 Department of Geriatrics, The Second Clinical Medical College of North Sichuan Medical College, Nanchong Central Hospital, Nanchong, Sichuan, China

**Keywords:** Neuroendocrine tumors, Lymph nodes, Prognosis, SEER, Propensity score matching

## Abstract

**Background:**

Current studies on the number of removed lymph nodes (LNs) and their prognostic value in small-bowel neuroendocrine tumors (SBNETs) are limited. This study aimed to clarify the prognostic value of removed LNs for SBNETs.

**Methods:**

SBNET patients without distant metastasis from 2004 to 2017 in the SEER database were included. The optimal cutoff values of examined LNs (ELNs) and negative LNs (NLNs) were calculated by the X-tile software. Propensity score matching (PSM) was done to match patients 1:1 on clinicopathological characteristics between the two groups. The Kaplan-Meier method with log-rank test and multivariable Cox proportional-hazards regression model were used to evaluate the prognostic effect of removed LNs.

**Results:**

The cutoff values of 14 for ELNs and 9 for NLNs could well distinguish patients with different prognoses. After 1:1 PSM, the differences in clinicopathological characteristics between the two groups were significantly reduced (all *P* > 0.05). Removal of more than one LN significantly improved the prognosis of the patients (*P* < 0.001). The number of lymphatic metastasis in the sufficiently radical resection group (SRR, 3.74 ± 3.278, ELN > 14 and NLN > 9) was significantly more than that in the insufficiently radical resection group (ISRR, 2.72 ± 3.19, ELN < 14 or NLN < 9). The 10-year overall survival (OS) of the SRR was significantly better than that of the ISRR (HR = 1.65, *P* = 0.001, 95% CI: 1.24–2.19).

**Conclusion:**

Both ELNs and NLNs can well predict the OS of patients. Systematic removal of more than 14 LNs and more than 9 NLNs can increase the OS of SBNET patients.

**Supplementary Information:**

The online version contains supplementary material available at 10.1007/s11605-021-04994-3.

## Introduction

Small-bowel neuroendocrine tumors (SBNETs) are a group of tumors originating from the neuroendocrine cells of embryos and having a neuroendocrine function. The incidence of SBNETs ranks the third among all NETs (after the lung and rectal NETs) and the first among all small-bowel tumors.^1^ A new study based on the Surveillance, Epidemiology, and End Results (SEER) database showed that from 1973 to 2012, the incidence of NETs increased by 6.4 times and the incidence rate of SBNETs reached 1.05/100,000.^2^ Some European studies have shown a similar growth trend.^3^ Surgery is currently the only treatment that can cure this disease. SBNETs are different from the NETs of the stomach and rectum, having a relatively high probability of metastasis^4^, ^5^ and relatively poor prognosis. Irrespective of regional lymph node metastasis (LNM), the major guidelines^6^, ^7^ recommend tumor resection combined with lymph node dissection (LND) of the mesentery and the root of the mesentery.^8^

As for other solid tumors, LNM is an independent poor prognostic factor for SBNETs.^9^ Simple LND may miss LN micrometastases with normal morphology, and the tumor cells remaining in the LNs could become the source of recurrence and metastasis. The level of LND that is sufficient for patient survival benefit has been defined in a variety of tumors.^10^, ^11^ After the radical resection of nonmetastatic SBNETs, the recurrence rate can be up to 42% at 10 years; emergency surgery, multiple tumors, and LNM are independent recurrence factors,^10^, ^11^ and LND in the drainage area can improve the survival of patients.^12^, ^13^ However, current studies on the number of removed LNs and their prognostic value in SBNETs are limited. In the 8th edition of the tumor, node, metastasis (TNM) staging manual by the American Joint Committee on Cancer (AJCC), the number of removed LNs is still not clearly defined, and the new N staging cannot distinguish between the prognoses of different patients. Some studies found that the percentage and number of positive LNs (PLNs) could be used as indicators of survival prediction,^9^, ^14^, ^15^ but this is still controversial. The total numbers of removed LNs and negative LNs (NLNs) can reflect the degree of LND in surgery and are independent prognostic factors in gastric cancer,^16^ rectal cancer,^9^ breast cancer,^17^ lung cancer,^18^ and others.^19^, ^20^ Whether the number of NLNs can be used as a prognostic indicator has been seldom reported for NETs, and whether more LNs removed improves the overall survival rate remains unclear.

Therefore, making use of the SEER database, we performed a retrospective analysis on the clinical data of SBNET patients without distant metastasis and with surgical resection between 2004 and 2017. The optimal numbers of examined LNs (ELNs) and NLNs were calculated by the X-tail program, and the prognostic effect of the number of removed LNs was investigated after using propensity score matching (PSM) to balance different clinicopathological factors.

## Methods

The pathologically confirmed SBNET patients (site code: C17.1, C17.2, and C17.3. ICD-O-3 histology code, 8240, 8246, and 8249) from 2004 to 2017 were collected from the SEER 18 database, which cover approximately 27.8% of all the American population, including information related to sociodemography and clinicopathology. The SEER*Stat 8.3.8 software was used to extract information from the database in our study, which was submitted in November 2019. This database is available for public cancer studies, and we have got the permission to obtain research data from the SEER database (Reference Number 11112-Nov2019). We have also promised not to identify any individual.

### Data

All patients with surgical resection of lesions (RX Summ-Surg codes 30, 40, and 60), aged 18 years or older, without multiple tumors and only M0 disease were included. In SEER database, codes 30 represented simple/partial surgical removal of the primary site, codes 40 represented total surgical removal of the primary site, and codes 60 represented partial or total removal of the primary site with an en bloc resection (partial or total removal) of other organs. Patients with unclear survival information, unknown exact tumor size, and unclear ELNs and NLNs were excluded from this study. Parameters, including tumor site and size, tumor extension, regional nodes examined, regional nodes positive, tissue type, surgical procedure, and survival status, were collected. Tumor grade according to the World Health Organization (WHO) classification was not available in the SEER database. Only tumor differentiation was retrieved. The TNM status of each patient was re-evaluated according to the 8th editions of the AJCC Cancer Staging Manual based on the tumor size, local extension, and LN involvement recorded in the SEER database.

### 2.2 Statistical Analysis

Continuous and categorical variables were expressed as mean ± SD and totals (percentages), respectively. The best cutoff values of ELNs and NLNs were calculated by the X-tile program in terms of OS.^21^ The chi-squared test or Fisher exact test or *t* test was used to quantify the differences between the two groups. The Kaplan-Meier method with log-rank test and multivariable Cox proportional-hazards regression model were used to evaluate the prognostic effect of clinicopathological factors. The above statistical analyses were performed with SPSS 22.0 (Chicago, IL, USA). *P* < 0.05 was considered statistically significant (two-tailed tests). The MatchIt package of the R software v3.6.3 was used to perform the 1:1 PSM with a caliper value set to 0.05 for different clinicopathological factors. The nearest-neighbor matching method was used to match the baseline characteristic differences between the two groups.

## Results

### General Condition

**Fig. 1:**
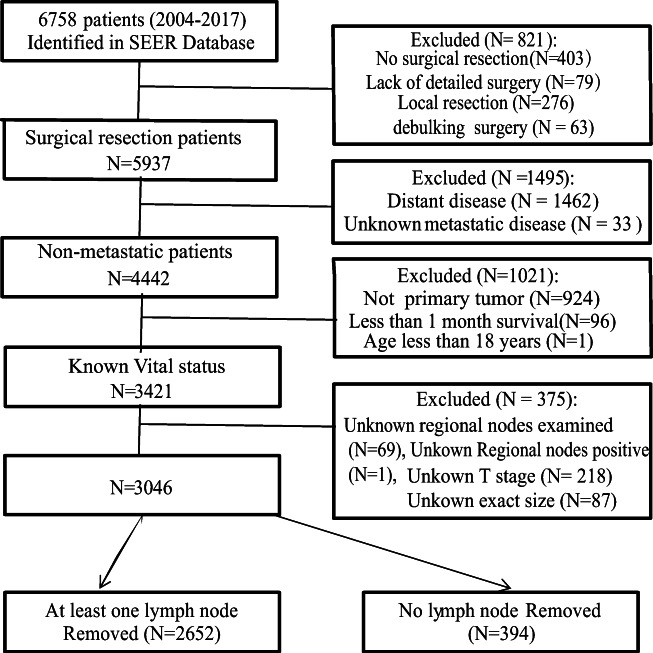
Flow chart of patients’ cohort selection

A total of 6758 pathologically confirmed SBNET patients were found in the SEER database. According to the exclusion criteria, 3046 SBNET patients were finally included. Among them, 2652 patients had more than one LN removed, and 394 patients had no LN removed (Fig. 1). Among the included patients, the sex ratio was approximately 1:1, 48.0% were older than 60 years old, and ileal NETs were much more common than jejunum NETs (89.7% vs. 10.3%). The main surgical approach was simple resection (2006 cases, 65.9%). The average tumor size is 18.4 ± 12.2 mm, and 10–50 mm was the most common (71.4%). The most common histologic type was carcinoid carcinoma (8246). Tumor differentiation was available in 2375 patients, and 74.7% of patients had a well-differentiated disease. LNM was found in 80% (2121/2652) of the patients. The last follow-up was November 2019, and the median follow-up period was 57.0 months. Detailed data are shown in Table 1.

**Table 1:** Distribution profiles of the clinicopathologic factors of the patients in the LND group and non-LND group before and after PSM matching

Characteristics	Cases (%) *N* = 3046	Before PSM			After PSM		
		Non-LND (*n* = 394)	LND (*n* = 2652)	*P* value	Non-LND (*n* = 365)	LND (*n* = 365)	*P* value
Age, years				< 0.001			0.55
≤ 60	1584 (52.0)	162	1422		161	153	
> 60	1462 (48.0)	232	1230		204	212	
Sex				< 0.001			0.822
Male	1520 (49.9)	164	1356		157	154	
Female	1526 (50.1)	230	1296		208	211	
Race				0.001			0.495
White	2609 (85.7)	313	2296		298	304	
Black	361 (11.9)	64	297		55	45	
Others	56 (1.8)	14	42		12	11	
Unknown	20 (0.7)	3	17		2	5	
Primary location				< 0.001			0.720
Jejunum	314 (10.3)	99	215		78	82	
Ileum	2732 (89.7)	295	2437		287	283	
Tumor size, cm	18.4 ± 12.2	13.3 ± 10.3	19.1 ± 12.2	< 0.001	13.7 ± 10.9	13.7 ± 7.1	0.949
Histologic type^a^				0.372			0.683
8240	2353 (77.2)	315	2038		289	281	
8246	615 (20.2)	71	544		68	73	
8249	78 (2.6)	8	70		8	11	
Surgery				< 0.001			0.500
Simple resection	2006 (65.9)	330	1676		301	294	
Partial resection	702 (23.0)	52	650		52	62	
Total resection	338 (11.1)	12	326		12	9	
Tumor differentiation				< 0.001			0.496
Well	1774 (58.2)	203	1571		194	184	
Moderate	422 (13.9)	37	385		35	51	
Poor	23 (0.9)	3	23		3	3	
Undifferentiated	2 (0.1)	1	2		1	1	
Unknown	671 (27.0)	150	671		155	161	
AJCC T status				0.01			0.834
T1	447 (14.7)	83	364		74	79	
T2	776 (25.5)	97	679		91	95	
T3	1215 (39.9)	147	1068		138	126	
T4	608 (20.0)	67	541		62	65	

*AJCC* American Joint Committee on Cancer, *LND* lymph node dissection, *PSM* propensity score matching

^a^International Classification of Diseases for Oncology, 3rd Edition (ICD-O-3): 8240, Carcinoid tumor; 8246, Carcinoid carcinoma; 8249, Atypical carcinoid tumor

### Comparison of Baseline Data and the Prognosis of LND Before and After Matching

**Fig. 2:**
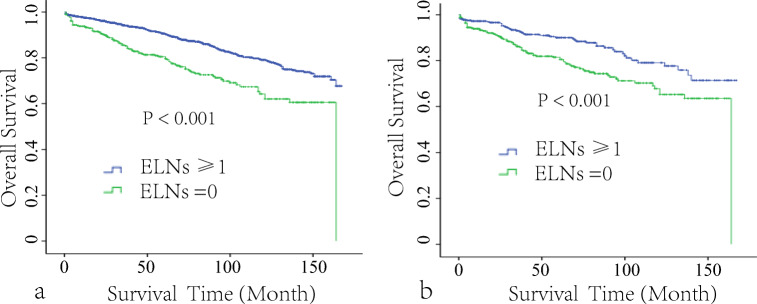
Kaplan-Meier survival curves of more than one lymph node (LN) removed and no LN removed before (**a**) and after (**b**) PSM matching. ELNs examined LNs

To verify the prognostic value of LND, this study enrolled 394 patients with no LN removed as the control group. Before the matching, survival analysis showed that removal of more than one LN significantly improved the 10-year OS (79.1 ± 1.3% vs. 64.2 ± 3.5%, *P* < 0.001, Fig. 2a); however, the patients of more than 60 years, female, jejunum NET, white race, simple resection, T1 stage, and well-differentiated disease in the no LN removed (non-LND ) group were significantly higher than in the more than one LN removed (LND) group (all *P* < 0.001) except for histologic type (*P* = 0.372). The mean tumor size in the non-LND group (13.3 ± 10.3 mm) was smaller than in the LND group (19.1 ± 12.2 mm, *P* < 0.001). PSM was used to balance the above significantly different clinicopathological factors. A total of 730 patients were selected according to the chosen 1:1 ratio, including 365 in each group. After PSM, the differences in clinicopathological characteristics were significantly reduced (Supplemental Figure 1), and none of the above factors was significantly different between the two groups after matching (Table 1). Survival analysis also showed that removal of more than one LN significantly improved the OS (79.1 ± 3.2% vs. 67.8 ± 3.5%, *P* < 0.001, Fig. 2b).

### Prognostic Role of ELNs and NLNs

**Fig. 3:**
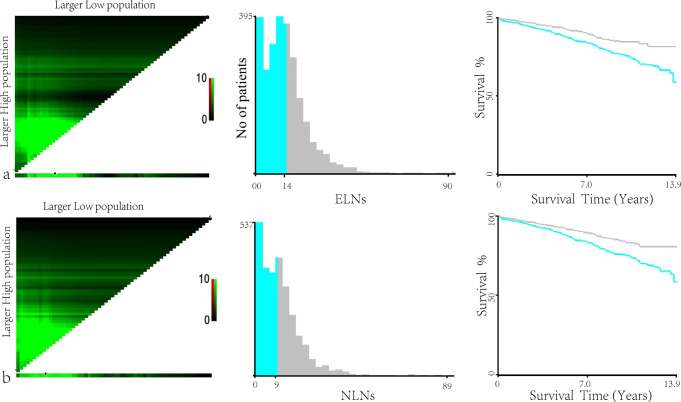
The optimal cutoff value of examined lymph nodes (ELNs), negative LN (NLNs), and its survival analysis. **a** X-tile analysis of survival data for ELNs, which divided the ELNs into low (≤ 14) and high (≥ 15) groups. **b** X-tile analysis of survival data for NLNs, which divided the NLNs into low (≤ 9) and high (≥ 10) groups

**Fig. 4:**
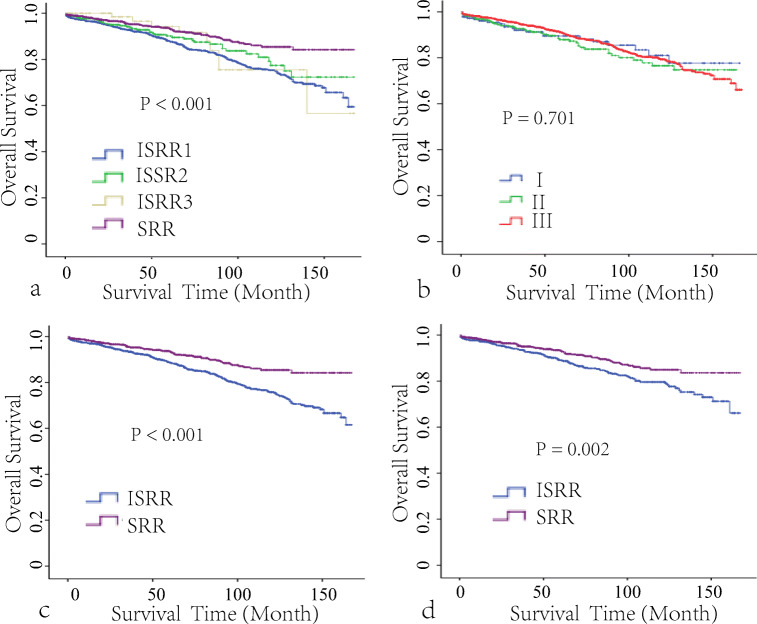
Kaplan-Meier survival curves of the four subgroups (**a**) according to the cutoff value of ELN and NLN: insufficiently radical resection (ISRR) 1 group (ELN ≤ 14 and NLN ≤ 9), ISRR 2 group (ELN ≤ 14 and NLN > 9), ISRR 3 group (ELN > 14 and NLN ≤ 9), and sufficiently radical resection (SRR) group (ELN > 14 and NLN > 9). Kaplan-Meier survival curves of the 8th AJCC TNM classification (**b**). Kaplan-Meier survival curves of SRR and ISSR (ELN ≤ 14 or NLN ≤ 9) before (**c**) and after (**d**) PSM matching. ELNs examined LNs, NLN negative LN

To investigate the effect of the number of lymph nodes removed on the prognosis, we further explored the ELNs and NLNs in the LND group. The X-tile showed that with OS as the endpoint event, the cutoff values of 14 for ELNs (Fig. 3a) and 9 for NLNs (Fig. 3b) could well distinguish patients with different prognoses. The higher the number of ELNs, the higher the OS (84.6 ± 1.8% vs. 75.8 ± 1.7%). The statistical results of NLNs were consistent with those of ELNs, and the 10-year overall survival rates were 83.2 ± 1.8% and 75.3 + 1.8% for the high and low groups, respectively. Then, we divided the LND group into four subgroups according to the cutoff value of ELN and NLN: insufficiently radical resection (ISRR) 1 group (ELN ≤ 14 and NLN ≤ 9), ISRR 2 group (ELN ≤ 14 and NLN > 9), ISRR 3 group (ELN > 14 and NLN ≤ 9), and sufficiently radical resection (SRR) group (ELN > 14 and NLN > 9). However, there was no significant difference in the survival rate among other groups except for the ISRR 1 group and SRR group (*P* < 0.001, Fig. 4a). To investigate the value of LND, we hypothesized that only the patient with ELNs greater than 14 and NLNs greater than 9 was considered sufficiently radical resection, namely SRR group; the remaining cases were in the ISRR group.

### Comparison of Baseline Data and the Prognosis of the Sufficiently Radical Resection and Insufficiently Radical Group Before and After Matching

Before the matching, age, primary location, tumor size, T stage, ELNs, and NLNs were also closely related to the prognosis of SBNET patients (Table 2, all *P* < 0.05), while gender, race, histologic type, surgery type, tumor differentiation, N stage, and TNM stage (Fig. 4b) were not associated to the prognosis (Table 2, all *P* > 0.05). The 10-year survival rates of the SRR group were significantly better than that of the ISRR group (85.4 + 1.9% vs. 75.8 + 1.6%, *P* < 0.001, Fig. 4c). However, the jejunum NETs, simple resection, well differentiation disease, and younger patients in the ISRR group were significantly higher than in the SRR group (all *P* < 0.001). The differences in race, sex, tumor size, and T stage were not statistically significant between the two groups (Table 3). To correct the differences in baseline characteristics between the two groups, PSM was used to balance age, tumor site, tumor differentiation, and surgery type. A total of 2048 patients were selected according to the chosen 1:1 ratio, including 1024 in each group. On this basis, the comparison of patients in the matched groups showed that the differences in clinicopathological characteristics were significantly reduced (Supplemental Figure 2), and none of the above characteristics was significantly different between two groups after matching (Table 3). After 1:1 PSM, the results showed that T stage, age, tumor size, tumor differentiation, ELNs, and NLNs were identified as potential prognostic factors (Table 2), and the number of PLNs in the SRR group (3.74 + 3.27) was more than in the ISRR group (2.72 + 3.19, *P* < 0.001, Table 3), while the 10-year survival rates of the SRR group (84.5 + 2.0%) were also better than that of the ISRR group (78.4 + 2.1%), which was consistent with before PSM. Multivariate analysis after adjusting for size, age, surgery, histologic type, and tumor differentiation showed that the HR of the ISRR group was HR = 1.65 (*P* = 0.001, 95% CI: 1.24–2.19).

**Table 2:** Survival analysis of the clinicopathologic factors in LND group before and after PSM matching

Characteristics	10 years		10 years	
	Before PSM (*n* = 2652)	*P* value	After PSM (*n* = 2048)	*P* value
Age, years		< 0.001		< 0.001
≤ 60	90.3 ± 1.2		90.7 ± 1.3	
> 60	65.5 ± 2.3		69.1 ± 2.8	
Sex		0.610		0.800
Male	78.0 ± 1.8		82.9 ± 1.9	
Female	80.4 ± 1.7		81.5 ± 1.9	
Race		0.651		0.727
White	79.1 ± 1.3		82.1 ± 1.5	
Black	78.8 ± 4.6		82.9 ± 3.5	
Others	74.7 ± 10.5		82.3 ± 10.3	
Unknown			-	
Primary location		0.023		0.989
Jejunum	66.8 ± 6.2		82.2 ± 1.4	
Ileum	80.1 ± 1.3		80.7 ± 7.1	
Tumor size, cm		< 0.001		< 0.001
≤ 1	85.2 ± 2.1		90.0 ± 2.0	
1–5	77.3 ± 1.6		79.8 ± 1.7	
> 5	62.9 ± 10.1		65.6 ± 9.8	
Histologic type^a^		0.249		0.071
8240	79.3 ± 1.4		83.1 ± 1.4	
8246	78.5 ± 3.4		78.1 ± 4.0	
8249	75.2 ± 12.6		65.5 ± 16.4	
Surgery		0.806		0.429
Simple resection	78.0 ± 1.7		83.0 ± 1.7	
Partial resection	81.6 ± 2.3		82.1 ± 2.5	
Total resection	79.9 ± 3.5		78.9 ± 3.9	
Tumor differentiation		0.102		0.040
Well	80.7 ± 2.1		85.0 ± 1.9	
Moderate	77.5 ± 3.6		75.6 ± 4.4	
Poor	58.3 ± 16.8		45.5 ± 32.4	
Undifferentiated			-	
Unknown	78..4 ± 1.8		80.9 ± 2.1	
AJCC T status		< 0.001		< 0.001
T1	88.4 ± 2.8		88.4 ± 2.8	
T2	87.0 ± 2.3		87.0 ± 2.3	
T3	79.8± 2.4		79.8± 2.4	
T4	75.3 ± 3.1		74.6 ± 3.6	
N stage		0.441		0.481
N0	77.7 ± 2.6		80.5 ± 3.0	
N1	79.3 ± 1.5		82.5 ± 1.5	
N2	86.3 ± 6.6		86.9 ± 7.0	
TNM stage		0.701		0.654
I	84.9 ± 42.1		86.8 ± 4.1	
II	74.7 ± 2.3		78.5 ± 4.3	
III	79.3 ± 1. 4		82.3 ± 1. 5	
ELNs		< 0.001		0.003
1–14	75.8 ± 1.7		80.0 ± 1.9	
≥ 15	84.6 ± 1.8		84.1 ± 1.9	
NLNs		< 0.001		0.004
1–9	75.3 ± 1.8		75.2 ± 1.9	
≥ 10	83.2 ± 1.8		84.4 ± 1.7	

AJCC American Joint Committee on Cancer, TNM tumor-node-metastasis, ELNs examined lymph nodes,NLN snegative examined lymph nodes

^a^International Classification of Diseases for Oncology, 3rd Edition (ICD-O-3): 8240, Carcinoid tumor; 8246, Carcinoid carcinoma; 8249, Atypical carcinoid tumor

**Table 3:** Distribution profiles of the clinicopathologic factors of the patients in the SRR and ISRR groups before and after PSM matching

Characteristics	Cases (%) 2562	Before PSM			After PSM		
		SRR (*n* = 1064)	ISSR (*n* = 1588)	*P* value	SSR (*n* = 1024)	ISRR (*n* = 1024)	*P* value
Age, years				< 0.001			1.0
≤ 60	1422	627	795		589	589	
> 60	1230	437	793		435	435	
Sex				0.477			0.757
Male	1356	553	803		535	528	
Female	1296	511	785		489	496	
Race				0.261			0.230
White	2296	919	1377		882	884	
Black	297	115	182		112	123	
Others	42	23	19		23	12	
Unknown	17	7	10		7	5	
Primary location				< 0.001			1
Jejunum	215	40	175		39	39	
Ileum	2437	1024	1413		985	985	
Tumor size, cm	19.1 ± 12.2	19.1 ± 12.4	19.1 ± 12.0	0.595	19.2 ± 12.6	19.5 ± 12.7	0.659
Histologic type^a^				0.897			0.456
8240	2038	813	1225		787	780	
8246	544	223	321		211	225	
8249	70	28	42		26	19	
Surgery type				0.001			1.0
Simple resection	1676	630	1046		629	629	
Partial resection	650	282	368		267	267	
Total resection	326	152	174		128	128	
Tumor differentiation				< 0.001			1.0
Well	1571	684	887		660	660	
Moderate	385	157	228		143	143	
Poor	23	6	17		6	6	
Undifferentiated	2	2	0		0	0	
Unknown	671	215	456		215	215	
PLNs	3.10 + 3.23	3.71 + 3.24	2.69 + 3.16	< 0.001	3.74 + 3.27	2.72 + 3.19	< 0.001
AJCC T status				0.158			0.389
T1	364	160	204		153	143	
T2	679	286	394		275	247	
T3	1068	407	661		388	413	
T4	541	212	329		208	221	

*AJCC* American Joint Committee on Cancer, *SRR* sufficiently radical group, *ISRR* insufficiently radical group, *PSM* propensity score matching

^a^International Classification of Diseases for Oncology, 3rd Edition (ICD-O-3): 8240, Carcinoid tumor; 8246, Carcinoid carcinoma; 8153, Gastrinoma; 8249, Atypical carcinoid tumor

## Discussion

LNM is the most common metastasis of gastrointestinal NETs. In this study, LNM was found in 80.0% of patients. This result was similar to that of other studies^4^,^5^ and was far higher than that found in other digestive tract tumors.^22^ Currently, for the treatment of SBNETs, the National Comprehensive Cancer Network^8^ and the European Neuroendocrine Tumor Society.^6^recommend the tumor resection combined with regional LND, and our results confirm that compared with the SBNET patients who had ≥ 1 LN removed, patients without LND had the worst prognosis. In 2006, the guidelines for SBNETs adopted the AJCC TNM staging system, but its actual application value is controversial. Strosberg 23 and Jann^24^verified the prognostic value of the current TNM staging system by a retrospective analysis, and the results unanimously showed that the current TNM staging system could only distinguish the prognosis of patients with metastasis (stage IV) vs. nonmetastasis (I–III) and that stages I, II, and III had similar prognoses. Our results were consistent with the above studies. The current 8th edition of TNM staging cannot distinguish the prognosis of SBNET patients with stages I to III.^25^

At the same time, the 8th edition of the AJCC Cancer Staging Manual defines N2 as PLNs ≥ 12, which requires more LNs to be removed to avoid underdiagnosing N2 as N1. However, the number and scope of LND have not been described in detail, and the prognostic value of LNM cannot be distinguished by the N staging of the 8th edition.^25^ Based on the above reasons, we studied the effect of the number of removed LNs on the prognosis, and the results revealed that ELNs and NLNs were closely correlated with prognosis. The patients with more than 14 ELNs or 9 NLNs had a significantly better prognosis than patients with fewer, and systematic removal of more than 14 LNs and more than 9 NLNs can increase the OS of SBNET patients.

Theoretically, the LNM behavior of SBNETs is similar to that of other tumors. Due to the possible existence of micrometastasis lymph nodes without dissection, conventional pathological examination of PLNs cannot fully reflect the LNM of SBNETs. Therefore, increasing the numbers of ELNs and NLNs within a certain range can ensure the thoroughness of the radical resection of SBNETs and obtain a good prognosis. A retrospective study by Chen et al.^25^ on 1925 SBNET patients in the SEER database between 2004 and 2014 showed that the removal of more than 12 ELNs significantly improved the prognosis. Zaidi 14 conducted a retrospective analysis of 119 SBNET patients with radical resection at multiple centers and found that at least eight LNs should be removed to accurately perform the TNM staging. There are differences between the results of this study and those of aforementioned studies. The studies of Chen et al.,^14^,^25^ included patients receiving local resection, which may lead to underestimation of the number of removed LNs, while this study only included the patients with pathological specimens after surgical resection. Differences in the disease severity of the included patients may account for the differences in the results.

In addition, the prognostic value of the PLN ratio in SBNET patients has also been reported,^26^ but if the number of ELNs is small, the PLN ratio is often overestimated and cannot truly reflect the actual state of the patient. As an important indicator of the degree of radical cure, NLNs are an independent prognostic factor in a variety of tumors, including gastric cancer,^16^ rectal cancer,^10^ colon cancer,^27^ and lung cancer^18^ but have been seldom reported in SBNETs. We demonstrated that NLNs can also be used as a prognostic indicator for SBNETs. The cutoff value of 9 can be used to classify NLNs into low and high groups, and the prognosis of the two groups is significantly different. Patients with NLNs > 9 had a good prognosis, while those with less than NLNs had a poor prognosis.

To better stratify the risk for SBNET patients, we divided the patients into four groups according to the cutoff value of ELN and NLN; however, this risk stratification can only distinguish ISSR 1 (ELN ≤ 14 and NLN ≤ 9) group and SSR group (ELN > 14 and NLN > 9) and has no discriminatory power for other groups. Therefore, only ELN greater than 14 and NLN greater than 9 were considered sufficiently radical resection in this study, and the other cases were divided into the ISRR group. The results showed that the prognosis of the SRR group was significantly better than that of the ISSR group; however, the potential prognostic factors, such as age, tumor site, surgical approaches, and T stage, were significantly different between the two groups. Therefore, we cannot infer that the survival benefit of patients in the SRR group comes from more lymph nodes removed.

Propensity matching score^28^ is a common and effective method when we are unable to do a prospective clinical study or the clinical study is low quality, which can simultaneously match the characteristics of multiple factors, minimize confounding bias, and better simulate clinical studies.^29^ Analysis based on a large sample size after PSM has more reference value. In our study, after the PSM, there was no significant difference in the clinicopathological distribution characteristics between the two groups, which improved the reliability of the conclusions of the subsequent analysis. As we expected, the prognosis of the SRR group was significantly higher than that of the ISRR group after balancing the clinicopathological factors. Furthermore, after adjusting potential prognostic factors, including age, T stage, and site, compared with the SRR group (ELNs ≥ 15 and NLN ≥ 10), the ISRR group had HRs of 1.653, indicating that removal of more than 14 LNs and ensuring at least 9 LNs could reduce the risk of death. Therefore, we believed that systematic removal of more than 14 LNs and more than 9 NLNs was the best choice, which can increase the OS of SBNET patients according to these results.

Like other studies based on the SEER database, this study also has its own limitations. First, there are many missing data that may lead to selection bias in the SEER database. Second, SEER data are from hospitals of different medical levels in 18 states of the USA, with differences in medical technology. Patients in high-level medical centers have better prognosis than those in general hospitals.^5^ Besides, independent prognostic factors, such as mesenteric masses and whether emergency surgery was done, are not provided in the SEER database, which may lead to overestimation or underestimation of the prognostic value of LNs. Although our data are not ideal, PSM performed a good balancing of the clinical and pathological characteristics of the two groups, reducing the selection bias. Of course, our results still need to be verified by prospective, multicenter, randomized controlled trials.

## Conclusion

This study was focused on the pathological confirmed SBNETs with surgical resection in the SEER database between 2004 and 2017, and the results showed that current TNM staging cannot accurately predict the survival of SBNET patients, and both ELNs and NLNs can well predict the overall survival (OS) of patients. Systematic removal of more than 14 LNs and more than 9 NLNs can increase the OS of SBNET patients.

## Supplementary Information

ESM 1 (PDF 219 kb)

ESM 2 (PDF 214 kb)
