# Supplementary figures and images for: Role of Removed Lymph Nodes on the Prognosis of M0 Small-Bowel Neuroendocrine Tumors: a Propensity Score Matching Analysis from SEER Database
Source: J Gastrointest Surg. 2021 Jun 9;25(12):3188–97. doi: 10.1007/s11605-021-04994-3 (PMC8654718; doi:10.1007/s11605-021-04994-3)

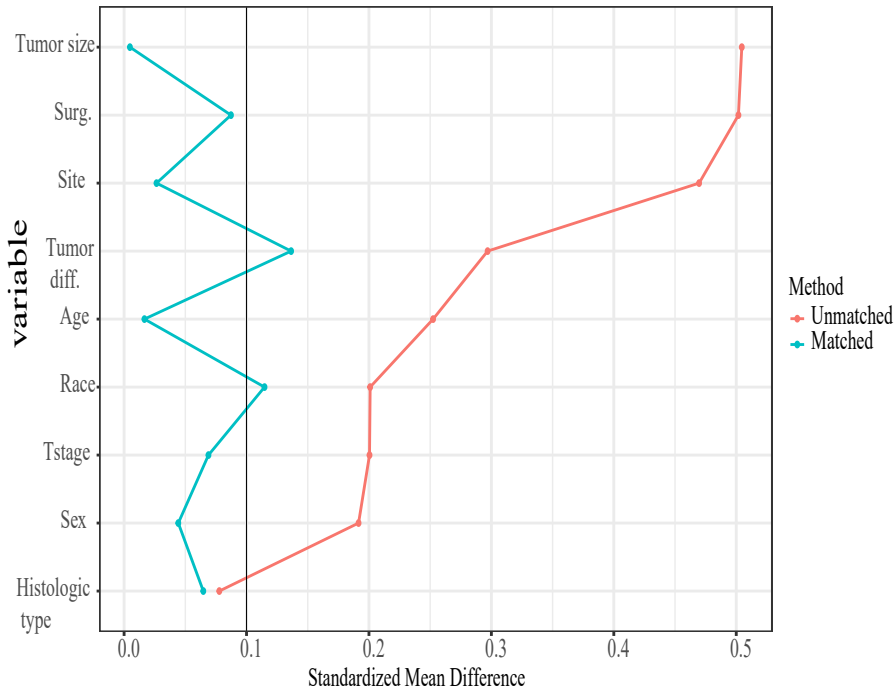

Supplement: Supplementary file 1 — (PDF 219 kb) [file 11605_2021_4994_MOESM1_ESM.pdf]

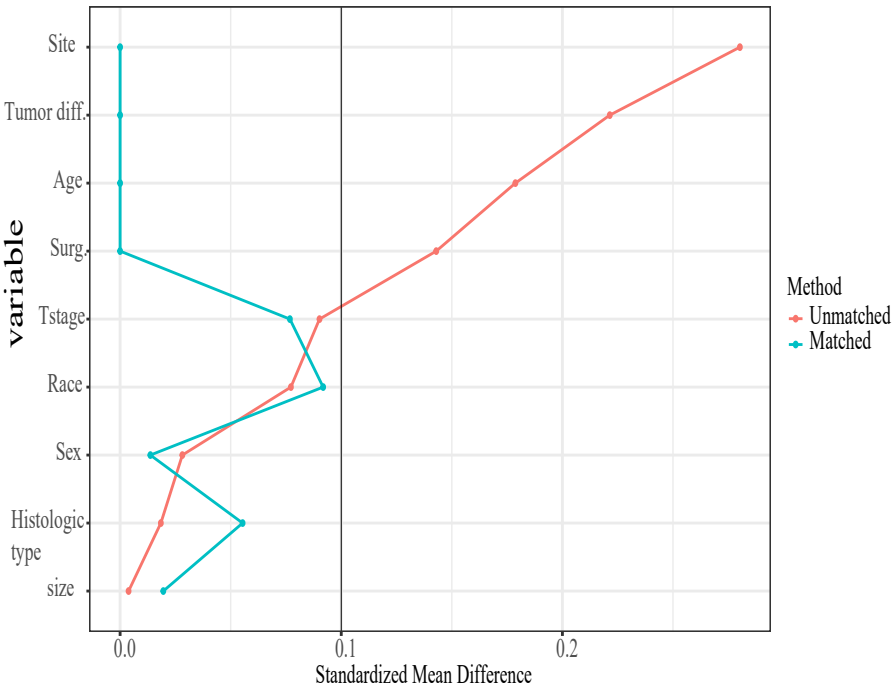

Supplement: Supplementary file 2 — (PDF 214 kb) [file 11605_2021_4994_MOESM2_ESM.pdf]
